# Redesigning printed educational materials for primary care physicians: design improvements increase usability 

**DOI:** 10.1186/s13012-015-0339-5

**Published:** 2015-11-04

**Authors:** Agnes Grudniewicz, Onil Bhattacharyya, K. Ann McKibbon, Sharon E. Straus

**Affiliations:** Institute of Health Policy, Management & Evaluation, University of Toronto, Health Sciences Building, 155 College Street, Suite 425, Toronto, Canada; Women’s College Hospital, University of Toronto, 77 Grenville St, Toronto, ON M5S 1B3, Office 217 Canada; Health Information Research Unit, Department of Clinical Epidemiology and Biostatistics, McMaster University Faculty of Health Sciences, Room 137 Communications Research Laboratory, 1280 Main Street West, Hamilton, ON L8S 4K1 Canada; Li Ka Shing Knowledge Institute, St. Michael’s Hospital, 209 Victoria Street, 7th Floor, East Building, Toronto, ON Canada

**Keywords:** Primary care, Educational materials, Usability, Design

## Abstract

**Background:**

Printed educational materials (PEMs) are a frequently used tool to disseminate clinical information and attempt to change behavior within primary care. However, their effect on clinician behavior is limited. In this study, we explored how PEMs can be redesigned to better meet the needs of primary care physicians (PCPs) and whether usability and selection can be increased when design principles and user preferences are used.

**Methods:**

We redesigned a publicly available PEM using physician preferences, design principles, and graphic designer support. We invited PCPs to select their preferred document between the redesigned and original versions in a discrete choice experiment, followed by an assessment of usability with the System Usability Scale and a think aloud process. We conducted this study in both a controlled and opportunistic setting to determine whether usability testing results vary by study location. Think aloud data was thematically analyzed, and results were interpreted using the Technology Acceptance Model.

**Results:**

One hundred and eighty four PCPs participated in the discrete choice experiment at the 2014 Family Medicine Forum, a large Canadian conference for family physicians. Of these, 87.7 % preferred the redesigned version. Follow-up interviews were held with a randomly selected group of seven participants. We repeated this in a controlled setting in Toronto, Canada, with a set of 14 participants. Using the System Usability Scale, we found that usability scores were significantly increased with the redesign (*p* < 0.001). We also found that when PCPs were given the choice between the two versions, they selected the redesigned version as their preferred PEM more often than the original (*p* < 0.001). Results did not appear to differ between the opportunistic and controlled setting. We used the results of the think aloud process to add to a list of end user preferences developed in a previous study.

**Conclusions:**

We found that redesigning a PEM with user preferences and design principles can improve its usability and result in the PEM being selected more often than the original. We feel this finding supports the involvement of the user, application of design principles, and the assistance of a graphic designer in the development of PEMs.

**Electronic supplementary material:**

The online version of this article (doi:10.1186/s13012-015-0339-5) contains supplementary material, which is available to authorized users.

## Background

Printed educational materials (PEMs) are an inexpensive and frequently used tool to disseminate clinical information and attempt to influence behavior change within primary care [1]. However, their effect on clinician behavior is limited [2]. As clinical evidence continues to grow faster than ever [3–5], it is likely that PEMs will continue to be used to disseminate evidence to clinicians, alone or as part of multi-component implementation interventions. Clinical practice guidelines, textbooks, and evidence summaries, among other forms of PEMs, are likely to continue to be offered in either electronic or printed versions. However, many of these PEMs are limited by poor design (i.e., do not follow design principles to present information in a useful and visually appealing way), which may make them less likely to be read by busy primary care physicians (PCPs). As such, it is important to optimize these tools and make them as useful and easy to use as possible to increase the likelihood that PCPs will use them in practice.

Improvement in the design of PEMs for PCPs may enhance their use [6]. Based on existing descriptions of PEM interventions, it appears that little attention has been paid to PEM design, likely due to resource limitations, limited design skills, and short timelines between when clinical practice guidelines are created and disseminated [6]. However, this lack of attention to design may be the reason why these materials have little to no effect. The effect of intrinsic characteristics such as design are demonstrated in the Technology Acceptance Model, a behavior change theory that posits that use of a product is based on perceived ease of use, perceived usefulness, attitude, and intention [7]. These factors are likely to be influenced by how a product (such as a PEM) looks and what information it contains. The Technology Acceptance Model can be used to understand the individual decision-making mechanisms that influence the adoption of new behaviors. It can also complement other implementation science frameworks such as the Knowledge to Action Cycle [8] that depict the process of distilling and implementing knowledge across contexts. Unlike the Diffusion of Innovations theory by Rogers [9], which focuses on the innovation being communicated, the Technology Acceptance Model examines the channel of communication for innovations such as clinical evidence, which is often disseminated in a one-way, linear method.

Design is not only a “cosmetic addition,” it is a requirement to facilitate interpretation and use of information. Visual cues are provided through design to enable a normal process of interpretation and understanding and poor design can limit the reader’s ability to decode information [10]. User-centered design supports the use of user preferences and of consultation with the user early and often in the design process. Following a user-centered design process and involving the end user in the design may increase the ability of the final product to meet user needs and goals and is advocated for use in health care technology to increase patient safety [11, 12].

Given their clinical responsibilities, PCPs have little time to devote to reading and studying new information relevant to patient care [13]. Studies show that PCPs feel overwhelmed by the amount of information available [5, 14] and by the amount of printed materials that cross their desk, delivered by email, mail, or fax [15, 16]. . Therefore, it may be worthwhile to optimize PEMs to increase the chance that PCPs will read, understand, and use them in their practice. In this study, we explored how PEMs can be redesigned to better meet the needs of PCPs and whether usability and selection can be increased when design principles and user preferences are used. We use the Technology Acceptance Model to frame our findings and understand the relationships between the end user’s perceptions of usefulness, ease of use, and their attitude and comments on intention to use the PEM.

## Methods

### Document redesign: selecting and redesigning an existing document

To study how an improved design may influence selection and usability by PCPs, we redesigned an existing, publicly available PEM. To select a tool to redesign, we identified tools that had been tested in RCTs. We selected a PEM that was included in a recent systematic review of PEMs for PCPs [Grudniewicz A, Kealy R, Rodseth R, Hamid J, Rudoler R, Straus S, The effect of printed educational materials on primary care physician knowledge, behavior, and patient outcomes: a systematic review and meta-analyses, Submitted] , the “Therapeutics Letter” [17]. In 2004, Dormuth and colleagues [18] tested the effect of 12 issues of the Therapeutics Initiative “Therapeutics Letter” on prescribing to newly treated patients. They found that when the effect of the 12 letters was combined, the probability of prescribing a drug recommended in the letter instead of another drug in the same class increased by 30 % in the 3 months after the letter was mailed. No single letter changed prescribing significantly on its own, but when combined, the positive change reached statistical significance. The Therapeutics Letters have been published regularly since 1994, with one to five letters published each year [17]. The Therapeutics Initiative uses a standard format across letters with a consistent layout and color scheme. Based on archived letters on the website, it appears that the design template has remained the same since the first letter was published in 1994. As such, we felt that the Letters showed potential for behavior change but had not yet been optimized. For this study, we used the most current document available at the time, Issue 89: Statins: Proven and Associated Harms from April/May 2014 (see Additional file 1). None of the authors of this paper are involved in the Therapeutics Initiative; we invited the coordinators of the Therapeutic Initiative to comment on the study; however, no comments were received.

Our redesign process was based on PCPs’ preferences gathered in a previous qualitative study[Grudniewicz A, Bhattacharyya O, McKibbon KA, Straus SE, User-centered design and educational materials: a focus group study of primary care physician preferences, Submitted] and design principles for the presentation of scientific information. We used a series of articles by Wong and colleagues [10, 19–37] as guidance for the redesign. We selected this series because it provides a synthesis of design principles and rules that are easy to apply for researchers outside the design industry to improve the presentation of scientific data. Only the rules applicable to the type of information presented in the Therapeutics Letter were used in our study. We complemented this set of design principles with user preferences as per user-centered design guidelines [38]. The PCP preferences are related to content and broader design concepts while Wong et al. focused more on data visualization and presentation of scientific information. Wong et al. recommended that the designer be familiar with professional design software, as inferior software and a lack of skills can hinder the ability to effectively communicate information [10]; thus, a graphic designer participated in the redesign of the document using Adobe Illustrator [39]. Working directly with the designer, AG redesigned the document by identifying and improving upon elements in the original letter that did not meet physician preferences and/or contradicted design principles and by employing additional design elements to match end user preferences. Several iterations of the document were reviewed by the study team, which included a PCP (OB). The PCP design preferences, Wong’s design principles, and designer contributions employed in the redesign are listed in Table 1. We did not add or change any content during the redesign, but some content deemed unnecessary was removed.

**Table 1:** Design preferences and principles used in the PEM redesign

Applied user preferences	Applied principles by Wong and colleagues [10, 19–37]	Designer contributions
Length	Layout	Software knowledge
- Page length kept to two pages	- Practical application of the golden section guide for grid layout	- Created document in Adobe Illustrator
- List of references on the third page removed (can be accessed online)	- Use of grid to determine placement of objects and text in order to build stability into the design	Simple design
- Paragraphs shortened by removing unnecessary words and breaking into shorter paragraphs	- Planning of the journey the reader’s eye will follow across the PEM to make it clear what is to be read first, second, etc.	- Helped create a simple and visually attractive design
Layout	- Framing of objects in ample white space to highlight importance	- Selected limited color scheme
- Content clustered into small groups	- Making objects visually different from others to highlight importance	Layout
- Content numbered where appropriate (see Advisories box)	Gestalt principles	- Applied golden section guide to create a grid layout for effective placement of text and objects
- Bolded and detailed headings that explain section content	- Grouping of objects by similarity and proximity	- Used white space to draw attention to important sections
Simple design	- Grouping objects with enclosures	Graphics
- Limited number of sections, graphics, and images	- Use of geometric shapes as alignment guides to create unified compositions	- Created stethoscope graphic to draw attention to the conclusions
- White space for visual appeal	- Using a grid to create alignment and help the reader identify patterns	- Created email and internet graphics to reduce text and increase visual appeal
- Limited color schemes	- Use of a grid to guide composition to create a clean and professional look	
- Clear division between sections	White space	
Visibility and accessibility of topic	- Use of white space to improve visual appeal and effectiveness of figures	
- Topic and title bold and clear at top of the PEM	- Enclosing images and text in “boxes” of white space to ensure good distribution of positive and negative space	
Key messages and highlighting of key points	- Use of small and large gaps of white space between sections to differentiate and group information	
- Key messages outlined below title	- Emphasizing important content with relatively more of available white space to attract the reader’s attention	
- Limited highlighting in text to ensure effect of main points is not reduced	Salience	
Text density and busyness	- Creating salience by using shape, color, and position on the page	
- Reduced clutter with spacing, bullet points, organized content, and structured layout		
- Removed unnecessary text to reduce text density		
Use of bullets and point form		
- Used bullets and point form instead of paragraphs where appropriate		
Color		
- Used color that prints well in black and white		
- Used color coding to match tables to text		
- Used color conservatively to maintain professional appearance and reduce distraction		
Font size		
- Attempted to make font as large as possible to ensure there was white space and content fit on two pages		
Logos and developing organization’s name		
- Included Therapeutics Initiative logo on a smaller scale at the top of the PEM		
- Included University of British Columbia logo at the end of PEM		
- Removed unidentifiable logo		

### Usability testing and a modified discrete choice experiment in two settings

Once the final version of the PEM was approved by the study team (see Additional file 2 for the redesigned document), we conducted usability testing on the original and redesigned versions and a modified discrete choice experiment with PCPs in two settings: an opportunistic setting and a controlled setting. Usability is “the measure of the ease with which a system can be learned and used,” within a specific context [40] and the more a tool or system helps a user find the information they need, the more usable it is [41]. Usability testing is the process of observing user’s interact with a system, document, or tool to identify human factor design flaws [38].

The opportunistic setting study was conducted over 3 days at a large Canadian conference for family physicians, the Family Medicine Forum in Quebec City, Quebec, in November 2014. This conference hosts approximately 3000 participants annually [42]. To determine the design preference of PCPs, a study author (AG) asked passing participants in the exhibit hall to select one of the two PEMs. The experiment was not advertised nor promoted with signage or in conference materials. The two PEMs were laminated and placed side by side on an otherwise empty table in an exhibit booth. The order of the PEMs on the table was rotated every hour. Participants were informed that the study was conducted for research purposes, and study information sheets were available. No other information was provided. Once participants selected their preferred version, we collected data on their gender, profession, and contact information for a follow-up usability test. Only data from PCPs and primary care residents were included in the analysis.

We aimed to follow-up with five to eight randomly selected participants from the conference in a telephone interview to conduct usability testing, as it has been found that as few as five participants can identify most usability problems [43, 44]. Recruitment was iterative and continued until thematic saturation was reached. Participants were emailed the PEMs immediately before the interview and were reminded which version they selected at the conference. The interviewer specified that she was not a part of the Therapeutics Initiative. During the interview, we asked participants to review the PEMs one at a time and “think aloud,” or verbalize their thoughts, by sharing their impressions and opinions [45]. The order of the PEMs was alternated for each interview starting with the original document at the first interview and rotating for each subsequent interview. Participants completed a usability test for each PEM immediately after its review. After the usability tests were completed, we asked participants to select their preferred version again, noting that they may select the same one or change their choice. We collected data on participant gender, age, years in practice, full- or part-time practice, affiliation, practice location (rural or urban), and practice model (group or single).

The experiment was repeated in a controlled setting to determine whether results were similar across the two settings. We invited PCPs practicing in the greater Toronto area, Ontario, Canada, to participate in 30-min in-person interviews either in their clinic or at St. Michael’s Hospital, Toronto. Participants were recruited via mass fax using a publicly available database from the College of Physicians and Surgeons of Ontario, the provincial medical licensing body. Recruitment letters were faxed to 1056 PCPs in December 2014. Though sample sizes for usability testing can be as low as two to five participants, five to eight participants are considered necessary for think-aloud processes [44] and as few as two for the System Usability Scale [46], we aimed for 10–20 participants to increase sample size for the modified discrete choice component. We continued to recruit until thematic saturation was reached during the think aloud process of the usability testing, and no new themes were being identified by the participants.

During the controlled setting interview, participants were shown both PEMs and asked to select their preferred version. They were then asked to carefully review one version of the PEM (with the order rotating at each interview, starting with the original document), with no other directions given to limit bias introduced by the interviewer. Participants were encouraged to think out loud and share their thoughts and opinions [45]. They then completed the usability test, and the process was repeated for the second version. Participants were asked to select their preferred version again after completing the usability test on both PEMs.

For all usability testing, we used the System Usability Scale [47] to measure the subjective usability of both PEMs. The System Usability Scale is a 10 item, validated questionnaire that uses a five-point Likert scale. It measures perception, is ideal for comparing across similar products, and does not take long to complete [47]. The scale has been applied to static documents by Kastner at al. [15] and Perrier et al. [48], and we similarly modified the wording of the scale slightly to apply to static documents by changing the word “system” to “document” (e.g., “I found this document unnecessarily complex,” “I thought this document was easy to use”), adding “content expert” to question 4 (“I think that I would need the support of a technical person or content expert to be able to use this document”), and providing examples in question 5 (“I found the various functions of this document (e.g., the tables, boxes, graphics, etc.) were well integrated”) (see Additional file 3 for full list of questions).

All telephone and in-person interviews were audio recorded and transcribed; transcripts were verified for accuracy. Interview participants received an honorarium.

### Data analysis

We calculated scores for the System Usability Scale (score out of 100); according to the scale’s guidelines, scores above 68 are considered above average and below 68 are below average [47]. Scores for each PEM across both settings were compared using a paired samples *t* test. Furthermore, we compared the number of participants that selected the original version to the redesigned version using a one sample chi-square test. Statistical analyses were conducted in SPSS version 22 [49].

We conducted an iterative, thematic analysis (as described by Braun and Clarke [50]) of the transcribed think aloud process in NVivo [51] to identify what elements participants liked and disliked about each PEM. We then used the Technology Acceptance Model to interpret the findings after analysis and explore the relationships between a PEM’s perceived ease of use and usefulness and the user’s attitude, intention, and actual PEM use [7]. Though intention and use were not explicitly measured, we explored themes identified in our analysis of the qualitative data to understand all the relationships presented in the Technology Acceptance Model.

The study was approved by the St. Michael’s Hospital Research Ethics Board and the University of Toronto Research Ethics Board.

## Results

### Opportunistic setting study: Family Medicine Forum

One hundred and eighty-eight clinicians from the Family Medicine Forum participated in our study; 184 of these were PCPs and primary care residents. Seventy-one percent of participants provided their email address for a follow-up interview. We contacted 36 randomly selected participants with an email invitation for a 30-min follow-up telephone interview. Invitations were sent out in three rounds until we reached thematic saturation. This was achieved with a sample size of seven participants [43]. Demographic information for all participants is presented in Table 2.

We compared the proportion of participants selecting the redesigned version to the proportion of participants selecting the original PEM at the Family Medicine Forum. The redesigned version was selected significantly more often (*n* = 162, 88 %) than the original (*n* = 22, 12 %), *χ*^2^ (1, *N* = 184) = 106.52, *p* < 0.001. Of the seven participants in the follow-up interviews, the mean System Usability Scale score was 44 for the original and 76 for the redesigned PEM. After the usability test was conducted, one participant changed their preference to the original PEM.

### Controlled setting study

Fourteen participants were interviewed in person as part of the controlled setting study; none of whom had been involved with the opportunistic setting study.

In the controlled setting interviews, 86 % (*n* = 12) of participants selected the redesigned version and 14 % (*n* = 2) selected the original, *χ*^2^ (1, *N* = 14) = 7.14, *p* = 0.008. The mean System Usability Scale score was 41 for the original and 71 for the redesigned version. After the usability test was conducted, three participants changed their selection from the redesigned to the original and the two participants that had selected the original PEM changed their selection to the redesigned version.

### Results across opportunistic and controlled setting

Across both settings and after completing the usability test, 81 % (*n* = 17) of participants selected the redesigned PEM and 19 % (*n* = 4) selected the original PEM as their preferred version. Across all interviews (*n* = 21), the System Usability Scale score was significantly higher for the redesigned version (mean 77.26) than for the original version (mean 45.71), *p* < 0.001.

Comparing the usability scores for each PEM across the two settings was done without inferential statistics as the small sample size would likely result in a type 2 error and would artificially support our hypothesis that there is no difference between the two settings. PEM preference was also similar across the two settings, with 86 % of participants preferring the redesigned version after usability testing in the opportunistic setting and 79 % of participants preferring the redesigned version in the controlled setting.

### Qualitative analysis

The qualitative analysis of the think aloud process is combined across both study settings. Content and format comments are presented by PEM version (see Table 3 for summary).

**Table 2:** Participant demographic characteristics

Category	Number	Percent (%)
Modified discrete choice participants, conference setting		
N	188	100
PCP or primary care resident	184	98
Other profession	4	2
Female	124	66
Email provided for follow-up	54	29
Interviewed participants, both settings		
N	35	100
Female	20	57
Age 25–35	13	37
Age 36–45	13	37
Age 46–55	8	23
Age 56–65	1	3
Years in practice <5	12	34
Years in practice 5–10	7	20
Years in practice 11–15	5	14
Years in practice 16–25	10	29
Years in practice >25	1	3
Full-time practice	25	71
Part-time practice	10	29
Academic affiliation	5	14
Hospital affiliation	4	11
Academic and hospital	9	26
No affiliation	17	49
Urban practice	34	97
Rural practice	1	3
Group practice model	33	94
Single practice	2	6

#### Original therapeutics letter: content

Some participants liked that the original version had more detail and better explanations than the redesigned PEM. They felt this was ideal for readers who want to understand the content well and have time to concentrate when reviewing the PEM. The original version was thought to be more thorough and the background that it provided was perceived to be a good refresher on the basics of the topic covered. Many participants noted that it “has this sort of journal feel to it” (C5:participant 5 from controlled setting), though this was said as both a positive and a negative comment, depending on the person. Participants that liked the detailed, journal-like style of the PEM commented that the narrative presentation of data was good, despite being “a little bit more intimidating at the beginning” (C10).

Participants felt it was difficult to identify the purpose and objective of the PEM and that the conclusions were vague and not applicable to clinical practice. Despite some participants liking the amount of detail presented, the majority of participants thought there was too much information in the PEM, making it overwhelming to read and use. One participant mentioned that “… it gets into a little bit too much data for people to quickly use… I’m reading it and I feel inundated …I’m not getting a clear picture, I feel like I’m a bit more confused” (O5:participant 5 from opportunistic setting). Another participant said: “…this document doesn’t isolate the important information and make it stand out” (C3). A common opinion was that any information that is not relevant to patient care should not be included, such as lengthy scientific introductions or detailed statistical data. Overall, participants agreed that the information was interesting but that it was not presented in a way that suited a busy PCP. This issue was most evident for statistical data, as most participants mentioned that many PCPs do not want numerical results such as confidence intervals and the absolute risk increase.

#### Original therapeutics letter: format

Several participants noted that the original version had strong visual appeal and stood out due to its bright colors, larger bolder font than the revised PEM, and good use of headings. Almost all participants found the PEM hard to read due to its dense prose, lack of bulleted text, and insufficient white space. The PEM was thought to be impractical for PCPs because it was hard to skim, was difficult to identify key points, took time to read, and would be difficult to reference in the future. One participant noted that “…in general practice… when we’re going through our day, and a document comes across our desk we’re usually skimming quickly” (O1), and another participant said that “I think something like this I’d be more apt to sort of ignore” (O1). The problem of too much information was exacerbated by poor layout. One participant said that this PEM is “…just not well formatted” (C10). Participants did not like that text columns were interrupted by large tables and that a large gray bar on the left of the PEM and a confusing graphic on the first page used significant space. A few participants mentioned that the graphic on the front page attracted their attention; however, the overwhelming majority of participants found it confusing because it had no title or legend and was not referenced in the text. Some found it distracting, childish, and impossible to decipher. Lastly, some participants disliked the PEM’s color scheme, saying the colors “don’t match” (O4) and that there were too many colors.

#### Redesigned therapeutics letter: content

Most participants commented first on the key messages at the top of the redesigned PEM. They felt that highlighted key messages work well with how they read PEMs by allowing them to see what the PEM is about and to easily decide if they want to read further. One participant said “I like that there are key messages rather than an introduction… just the first few dot points that tell me what I’m going to be learning… sort of why I am going to read it” (O7). They liked that the purpose of the document was made clear at the start and that key messages were short and bulleted. Similarly, the bulleted conclusion was liked as it was specific, actionable, brief, clear, and easily found.

Some participants thought that the content was not specific enough and wanted the key points to be less vague and to contain more information on patient care, saying “…it would be nice if maybe there was a little box…. Instead of the tables, use your space to maybe put in the approach to dealing with patients… because that’s practical” (C14).

#### Redesigned therapeutics letter: format

Some participants who reviewed the redesigned version after the original commented immediately that the redesigned PEM was much easier to read and less intimidating on first glance, saying “I already like it better…” (O4), “…already I feel less stressed” (O6), or “Okay. A little more attractive” (C11). The PEM was considered visually appealing with good use of white space, light colors, and small graphics used to highlight key areas. However, some participants said they did not like the smaller font that was used in the PEM, in particular, in the tables. Participants felt it would be better to reduce the white space and enlarge the font size.

Participants found that it was easy to skim or scan the PEM and identify key information due to the layout, bullet points, point form, color coding that matched the text to the tables, and less information. However, a few participants were confused that a box on “Why Aren’t the Harms of Statins More Commonly Acknowledged?” was placed below the conclusion and felt this was poor layout. A few participants noted that the flow and layout of the PEM could be improved, saying “somehow it just doesn’t seem to, perhaps, flow as naturally as one might desire” (C8).

Tables were found to be a little easier to read in the revised PEM, with fewer numerical results. Participants liked that all the tables were on the front page and that boxes were used to display additional information. Overall, many participants felt the PEM would be more useful in practice, with one participant saying “I’d be shocked if this is not the one that people overwhelmingly prefer” (O6).

#### Content and format across both versions

There were some comments that applied to both versions of the PEM. Participants felt that more data should be provided on how to apply the information to patient care as this was the most useful information for PCPs. They noted that the tables of harms should be organized by magnitude. Further, they felt that statistical data are difficult to quickly interpret for a PCP.

## Discussion

We redesigned a recent version of the Therapeutics Letter with user preferences and design principles, applied with the support of a graphic designer. Using the System Usability Scale to measure perceived usability, we found that scores were significantly increased with the redesign. We also found that when PCPs were given the choice between the redesigned version and the original, they selected the redesigned version as their preferred PEM more often than the original. Twelve previous issues of the Therapeutics Letter had a significant cumulative effect on prescribing behavior; however, no single letter had a significant effect on its own on prescribing [18]. Based on our results, there is potential for redesigned Therapeutics Letters to be perceived as more useful and easy to use by readers, which may increase their use and potentially their effect on physician behavior.

In our qualitative analysis of the think aloud process in the usability test interview, we found several points of strong agreement and several points of contention across participants. There was strong support for the use of bullet points and point form within PEMs. All participants commented on the usefulness of the key messages listed in bullets at the top of the redesigned version. They also agreed on the importance of actionable conclusions that are relevant to primary care practice. Participants preferred ample use of white space to create an attractive PEM that is not overwhelming and use of headings and highlighting of key areas to facilitate content skimming and future reference. Lastly, many participants commented on the use of boxes for additional information as this is a familiar format used in journals and creates an easy to navigate layout.

There was less agreement on the use of narrative and prose among participants. Though most participants liked bullets and point form text, several participants were drawn to the prose in the original version. These participants felt that if they had the time to review the PEM carefully, a more comprehensive narrative overview akin to a journal would be more useful. However, context may have influenced this perspective. As not all participants were interviewed within their busy office settings, their opinions during the interview may differ from preferences they have while working in a busy clinic. The provision of numerical results was also a point of contention for each participant. Many noted that they do not have the time or skills to interpret complex numerical data and that they either struggled to understand the tables or skipped over them. However, they also noted that without tables and numerical results, the PEM may lose credibility. Ideally, numerical results should be included in formats that are easy to understand such as number needed to treat/harm and dot diagrams (where one point represents one patient in a chart or graphic) or other easy to interpret representations of data. Tables of numerical results of studies should not take too much space and should always be paired with a written interpretation within the text of the PEM with a clear link between the text and tables.

A second point of contention was visual appeal. Some participants liked the bright colors and abundant highlighting present in the original version. Others preferred the lighter colors of the redesign. This appears to be a stylistic preference and a balance between very bright and very soft pastel colors appeared to be the preference among our participants; a professional graphic designer may be useful in achieving this balanced effect. Our participants also preferred the use of a standard color scheme across publications to allow for branding and user recognition of the product. Visual elements such as pictures and graphics were also very contentious, and the abstract graphic in the original version sparked strong opinions. When graphics are small, simple, and draw the eye to an important area such as key messages or conclusions, they seem generally well accepted. Abstract, unidentifiable, and unlabeled visuals should be avoided.

The results of this study can be interpreted in light of the Technology Acceptance Model. In order to increase PCPs’ use of PEMs, the Technology Acceptance Model theorizes that perceived ease of use and perceived usefulness need to be increased. These perceptions will then improve end user attitude toward using PEMs, creating behavioral intention, and may ultimately influence system use. Our results support the relationship between perceived ease of use, perceived usefulness, and attitude toward the PEM. We measured ease of use using the System Usability Scale and collected data on the usefulness of the redesigned and original document. By asking participants to select their favorite of the two versions, we attempted to capture attitude and the first steps of behavioral intention toward using the PEM. Participants directly commented on ease of use as a function of the design and found that the version that was more easy to use would be more useful within their practice. The easier to use and the more useful the PEM, the more positive their attitude was toward the PEM. We also attempted to measure attitude and behavioral intention to use the PEM with our modified discrete choice experiment where we asked participants to select their favorite version. Though this was not a direct measure of intention, we felt it served as a preliminary indication of intention and found that the redesigned version was selected more often both before and after the usability test. The results support the relationships between perceived ease of use, perceived usefulness, and attitude, suggesting that poor PEM design may lead to low use of PEMs by PCPs by affecting end user attitude and intention.

User-centered design encourages early and frequent involvement of the user throughout the design process [38]. However, due to time and resource limitations, it may not be feasible to involve the user throughout the entire PEM design process. Therefore, we created a list of key design elements for the design of PEMs for PCPs, presented first in an earlier version in our focus group paper, and expanded upon with results from this study (see Table 4). We recommend that these PCP preferences be used as a starting point in the design process, paired with design principles such as those outlined by Wong and colleagues (see Table 1). We also recommend the support of a graphic designer to apply and balance design principles with user preferences, though a graphic designer cannot replace the collection and application of end user preferences. The design principles presented by Wong et al. complement the list of user preferences; Wong’s design principles refer to the more specific elements of design and information presentation while the user preferences we collected are related to general content and format specifically relevant to the primary care context.

**Table 3:** Summary of qualitative analysis of think aloud process

	Content	Format
Original PEM	Lots of Detail - Thorough - Good background information - Nice narrative Too Much Information - Information that is not relevant to patient care - Too much information in tables - Overwhelming - Too much introductory/background information Purpose Not Clear - Objective of PEM not specified Vague Conclusions - Vague and not actionable in practice Tables - Too much statistical data - Not clear	Hard to Read - Too much text - Dense prose - Difficult to skim - Difficult to pick out key points - No bullets - Requires concentration to read - Hard to use in practice Unorganized Layout - Poorly formatted - Tables disrupt flow of text - Insufficient white space Tables - Cluttered - Inconsistent formatting Graphic on First Page - No legend, title, or explanation - Confusing - Childish - Distracting from the information - However, - Draws attention to the PEM - Artistic Color - Colors do not match - Too many colors, too busy Visual Appeal - Bright and colorful - Attracts attention - Larger font - Good use of headings
Redesigned PEM	Key Messages - Three easy to read bullets - Make purpose of PEM clear - Helpful for quick reference Conclusion - Useful - Brief and clear Not Specific Enough - More specific dosing information - No information on patient care - Key messages too vague Tables - Tables difficult to understand - Statistical data difficult to interpret Layout - Box on harms below conclusion was confusing Useful in Practice	Tables - Rows not organized by magnitude of harms - Tables difficult to understand - Text too small Layout - Lacking flow from section to section Visually Appealing - Use of white space - Use of soft colors - Not visually overwhelming - Small graphics (stethoscope) used to highlight key areas Easy to Read - Easy to identify key information - Easy to skim or scan PEM - Uses bullet points and point form - Layout is easy to follow - Color coding used to match text to tables - Not too much information, less complex - Tables have fewer, easier to interpret statistics Layout - Use of boxes for additional information - Tables all on one page - Division of text by study type Small Font Useful in Practice

**Table 4:** Primary care physician preferences for the design of printed educational materials

Design element	Details
Length	- One to two pages, maximum
	- Short paragraphs or bullet points and point form sentences*
Layout	- Numbered clusters preferred over paragraphs
	- Two columns preferred versus one column, when appropriate
	- Single sided pages preferred by some physicians to make it easier to post materials on the wall or on bulletin boards
	- Bolded and detailed headings that explain the content of the following section facilitate finding the right information and help physicians decide if they are interested in that section*
Simple design	- Simple designs attract the user to the PEM*
	- Limited sections, graphs, and images*
	- Use of white space*
	- Limited color schemes that are neither too bright and overwhelming nor too light and pale*
	- Clear division between sections with the use of headings*
Visibility and accessibility of topic	- Topic and title should be bolded and clear*
	- Bolded topic and title help the reader decide if the content is relevant and of interest*
Key messages and highlighting of key points	- Main messages outlined at the top of the PEM*
	- Clearly outlined goals*
	- Key information highlighted to stand out from the rest of the text*
	- Over-highlighting can reduce the effect of emphasizing main points
Text density and busyness	- Overly busy materials may be discouraging to the reader*
	- Busyness can be reduced with use of white space, good organization of content, spacing between lines and paragraphs, bullet points, flow-charts, numbering, and a structured layout*
	- Too much text can reduce information recall*
	- Text-heavy PEMs reduce information retrieval and make it more difficult to scan for information*
	- Too much information on a PEM makes it hard to use in practice*
	- Electronic materials are more difficult to read on small screens if text-heavy
Use of bullets and point form	- Bullets and point form are preferred over paragraphs and full sentences as they facilitate quick reading*
	- FAQs (including the answers) work best in point form
Color	- Color is preferred and can be used to organize text*
	- Color can draw the eye and attract the reader to the PEM*
	- Color PEMs should print and photocopy well in black and white
	- Color can influence credibility and too much color can reduce the perception of credibility*
	- Too much color or colors that are too bright can compete with text and be distracting*
	- Color coding can be used to match text to tables or charts*
Font size	- Small print discourages reading (ideal size can be determined through cyclical usability testing)*
	- Larger print should be prioritized over ample white space*
Logos and developing organization’s name	- Logos should be used to show who has developed the materials
	- Use of logos can increase the perception of credibility
	- Logos are best placed at the top of materials, should be used sparsely, and need to be recognizable
	- Unrecognizable logos should be paired with the name of the organization
Templates and common formats	- Use of recurring formats across materials by the same organization facilitates navigation of the PEM*
Use of graphics, images, or other visuals	- Graphics should be labeled, be referenced in text, and use legends when appropriate*
	- Small images can be used to draw attention to an important area such as conclusions or clinical implications*
Tables	- Should not contain difficult to interpret numerical results such as risk ratios and odds ratios*
	- Use large font to make tables easy to read*
	- Use white space to make tables attractive and less intimidating*
Specificity	- Content should be specific enough to use in practice and not require looking up further information*
	- Conclusions and key messages need to be very specific*
	- Vague comments should be avoided*

*Preferences that were confirmed or added as a result of this study

Few studies have examined the design of PEMs and the relationship between design and usability. Our results are supported by limited qualitative studies that found that PCPs want more user-friendly evidence-based materials [52] and that summaries of guidelines should be written in clear and actionable language [15]. A review of the literature by Versloot and colleagues [6] also concluded that format influences accessibility and usability. The authors identified ways to apply formatting principles similar to those discussed in this study to influence the usability of guidelines. Our study builds on the existing literature on barriers and enablers of guideline uptake by collecting primary data from the user and testing how the redesign process can influence usability. We provide a practical, detailed, and actionable list of elements that can be used to design PEMs with support from a graphic designer. Similar guidance to the creation of PEMs has been developed by the US Centers for Disease Control and Prevention, which lends support to our list of PCP design preferences [53]. However, we provide expanded details relevant to PCPs, including page length, simple and attractive design, PEM goals, text busyness, color, and logos. The results of this study can support efforts to optimize PEMs to make them as useful and easy to use as possible. By applying our PEM development process to improve and optimize PEMs, their usability can be increased, which may increase the likelihood that PCPs will select, read, and use them in practice.

This study has some limitations. As with all qualitative studies, it is limited in its generalizability. Our opportunistic setting study at the Family Medicine Forum was limited to Canadian PCPs. Our usability test included a small sample of participants that were self-selected and may have a stronger interest in educational materials than the general population. Rural physicians and those in single practices were under-represented in our sample. However, our sample included participants from across Canada and from various primary care settings and age groups. Our results are also based on participant perceptions, and we did not examine behavior in clinical practice. As participants were asked to carefully read the PEMs during the usability testing, they were likely to pay more attention to certain features and content than they may have otherwise done in practice. We attempted to replicate a setting where participants would be choosing PEMs for future reading by conducting our modified discrete choice experiment in a large exhibit hall at a national conference. Furthermore, the results of this study are based on the redesign of only one PEM and may not be generalizable to the redesign of other PEMs. However, user preferences applied in this study were based on a previous focus group study that addressed design preferences more generally.

Lastly, user-centered design suggests involvement of the user throughout the entire design process and usability testing should be best done in a cyclical format rather than once. However, this was not possible due to limited resources, and we felt it was not a necessary step in answering the research question as five to eight participants has been shown to find 80 % of usability problems [44]. In practice, involvement of the user throughout the design process and several iterations of usability testing to gather user feedback is recommended [38]. However, our results show that even a single cycle of usability testing provides value.

## Conclusions

We found that redesigning an existing PEM with user preferences and design principles can improve its usability and result in the PEM being selected more often than the original. We feel that this finding merits further research and the investment of time and resources into better design of PEMs, specifically the involvement of the user, application of design principles, and the assistance of a graphic designer. Once PEMs are optimized, further studies could determine if optimized PEMs can increase knowledge and influence practice.

## Additional files

Additional file 1:
**Original Therapeutics Letter Issue 89: Statins: Proven and Associated Harms, April/May 2014.** ᅟ Additional file 2:
**Redesigned Version of the Therapeutics Letter.**ᅟ Additional file 3:
**Modified System Usability Scale.** (DOCX 13.3 KB)

## Abbreviations

PCP: primary care physician
PEM: printed educational materials
