# Supplementary material for: Redesigning printed educational materials for primary care physicians: design improvements increase usability
Source: Implement Sci. 2015 Nov 4;10:156. doi: 10.1186/s13012-015-0339-5 (PMC4634785; doi:10.1186/s13012-015-0339-5)
Supplement: Additional file 1: — Original Therapeutics Letter Issue 89: Statins: Proven and Associated Harms, April/May 2014. ᅟ [file 13012_2015_339_MOESM1_ESM.pdf]

therapeutics letter©

therapeutics letter
April - May 2014

THERAPEUTICS INITIATIVE Evidence Based Drug Therapy

Statins
proven and associated harms

Therapeutics Letter #49 (Jul-Sept 2003) concluded that "Statins provide a cardiovascular and total mortality benefit for patients with clinically evident occlusive vascular disease (secondary prevention)" and Letter #77 (Mar-Apr 2010) concluded that "Statins do not have a net health benefit in primary prevention populations", because they reduce coronary heart disease (CHD) serious adverse events (SAEs), but have no effect on total SAEs. This suggests that there are unidentified SAEs caused by statins that counterbalance the reduction in CHD SAEs.

Concerns about SAEs related to HMG-CoA reductase inhibitors (statins) were first raised in 2001, when cerivastatin was withdrawn from the market after being linked to over 100 deaths from muscle damage occurring at a rate much higher than other statins.[1]

This Letter examines proven and associated harms with statin use. Proven harms are those that have been established in systematic reviews or randomized controlled trials (RCTs); associated harms are those identified from observational studies, case series and case reports.

How do statins work?

Statins inhibit the enzyme 3-hydroxy-3-methylglutaryl-coenzyme A (HMG-CoA) reductase at an early stage of the mevalonate pathway. Cholesterol is generated by this pathway, but so are a number of other products with a pivotal role in bodily functions, such as coenzyme Q10, heme A, isoprenylated proteins, sex steroids, corticosteroids, bile acids, and vitamin D.[1] Statins reduce plasma cholesterol by inhibiting HMG-CoA reductase in the liver, but also inhibit this enzyme in tissues throughout the body.

Why aren't the harms of statins more commonly acknowledged?

First, most of the literature on statins has focused on the benefits. As a result awareness of statin harms is low [2], and many specialists propound that statin harms are very unusual.[3] Second, the reported incidence of common statin effects, such as muscle pain and weakening, is low in randomized trials but higher in studies of real world use.[1,4] To some extent this is explained by use of a 'run-in period' in some statin RCTs when all patients are exposed to the drug prior to randomization and only those tolerating the drug are randomized.[5] Third, warnings about statin-related harm issued by the US FDA or Health Canada are slow to be released, and as with past advisories have little impact.[6]

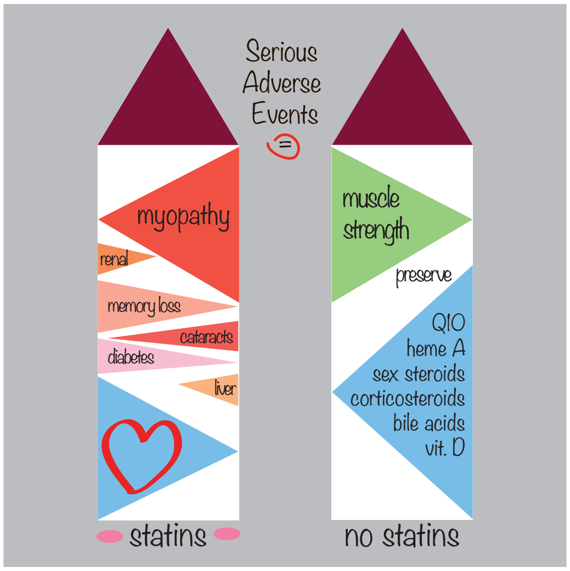

Canadian and US Advisories on statin-related harms

Since 2000 Health Canada has issued five ADR advisories related to statins: rhabdomyolysis and myopathy (January 2002),[7] Crestor and rhabdomyolysis (November 2004),[8] existing medical conditions which may increase risk of statin-related muscle problems (July 2005),[9] statins and memory loss (October 2005),[10] and statins and interstitial lung disease (October 2010).[11] On February 28, 2012, the US FDA revised statin labels, warning of the potential for "generally non-serious and reversible cognitive side effects (memory loss, confusion, etc.) and reports of increased blood sugar and glycosylated hemoglobin (HbA1c) levels".[12]

Proven statin harms

Highest level of evidence: Systematic Reviews

The magnitude of harms from meta-analyses of RCTs are shown in Table 1.

Table 1. Statin harms demonstrated in meta-analysis of RCTs

Outcome	Statin Dose	RR or OR [95% CI]	ARI NNH	Ref.
Withdrawal due to adverse effects	High*vs low#	1.3 [1.2, 1.4]	2.1% 47 (3.4 yr)	13
Muscle damage (CK elevation >10x normal)	High*vs low#	10.0 [1.3, 78.0]	0.07% 1534 (3.4 yr)	13
Liver enzyme elevation	High*vs low#	4.8 [3.3, 6.2]	1.2% 86 (3.4 yr)	13
Newly diagnosed diabetes	High*vs low#	1.12 [1.04, 1.22]	1.0% 105 (4 yr)	14
Newly diagnosed diabetes	All doses	1.09 [1.02. 1.17]	0.4% 250 (4 yr)	15

* High dose - simvastatin 80 mg, atorvastatin 40-80 mg.
\# Low dose - simvastatin 20 mg, pravastatin 40 mg, atorvastatin 10 mg.
RR- relative risk, OR- odds ratio, ARI- absolute risk increase, NNH- number of people needed to treat to harm one person.

Mailing Address: Therapeutics Initiative
The University of British Columbia
Department of Anesthesiology, Pharmacology & Therapeutics
2176 Health Sciences Mall
Vancouver, BC Canada V6T 1Z3

Tel.: 604 822•0700
Fax: 604 822•0701
E-mail: info@ti.ubc.ca
www.ti.ubc.ca

Second level evidence: single RCTs

Some statistically significant statin harms have been demonstrated in large RCTs. Others have been demonstrated in smaller RCTs designed to measure a specific effect: e.g. the 2012 RCT showing that simvastatin and pravastatin significantly decrease energy and increase fatigue after exertion compared with placebo[16] and the 2013 RCT demonstrating that simvastatin significantly attenuates cardiorespiratory fitness as compared to placebo in overweight and obese patients.[17] (See Table 2).

Table 2. Statin harms demonstrated in single RCTs

Outcome	Statin Dose	RR or OR [95% CI]	ARI NNH	Ref.
Hemorrhagic stroke	High*	1.7 [1.1, 2.6]	0.9% 108 (5 yr)	18
Cognition	Low	Decrease		19,20
Energy<br>Fatigue with exertion	Low	Decrease<br>Increase		12
Cardiorespiratory fitness	Low	Decrease		13
Sleep quality	Low	Decrease		21
Newly diagnosed diabetes	High#	1.25 [1.05, 1.49]	0.6% 167 (1.9 yr)	22

*atorvastatin 80 mg, #rosuvastatin 40 mg.

Associated statin harms

Third level evidence: Observational studies

The magnitude of statin harms has also been estimaed in large observational studies (see Table 3).

Table 3. Statin harms supported by large observational studies

Outcome	Statin Dose	RR or OR [95% CI]	ARI NNH	Ref.
Acute kidney injury	High* vs low	1.34 [1.25, 1.43]	0.06% 1700 (0.25 yr)	23
Acute renal failure	All doses vs no statin	1.6 [1.3,1.9]	0.2% 450 (5 yr)	24
Moderate or serious liver dysfunction	All doses vs no statin	1.5 [1.4, 1.7]	0.7% 150 (5 yr)	24
Moderate or serious myopathy	All doses vs no statin	6.2 [5.2, 7.3]	1.0% 100 (5 yr)	24
Musculoskeletal conditions	All doses vs no statin	1.19 [1.08,1.30]	2.1% 48 (4.4 y)	25
Cataracts	All doses vs no statin	1.3 [1.26, 1.37]	2% 50 (5 yr)	24

* ≥ 10 mg rosuvastatin, ≥ 20 mg atorvastatin, ≥ 40 mg simvastatin

Fourth level evidence: Case series and case reports

A longer and growing list of harms is supported by case series and case reports. These are documented and referenced in the detailed analysis by Golomb and Evans and include peripheral neuropathy, sexual dysfunction, gynecomastia, irritability, aggression, behaviour change, memory loss, depression, psychosis, interstitial lung disease, heart failure, Parkinson syndrome, lupus-like syndrome, dermatomyositis, other auto-immune syndromes, pancreatitis and others.[1]

For the complete list of references go to **www.ti.ubc.ca/letter89**

Muscle symptoms, the commonest statin adverse effect

Patients taking statins can experience muscle pain, aches, soreness, weakness or fatigue, but these symptoms also occur in people not taking statins.[4] An approach to dealing with patients with statin related muscle symptoms is provided by Fernandez et al.[26] It is important to appreciate that falls in the elderly could be due to statin adverse effects on muscle. The incidence of muscle symptoms is low in RCTs[4] but higher in observational studies.[27] Muscle symptoms interfering with exercise and inhibition of cardiorespiratory fitness[17] are problematic because regular exercise is the best way for patients to prevent adverse cardiovascular events.[28] Minor muscle damage may be very prevalent as low level ultrastructural muscle damage was detectable in muscle biopsies from 10 of 14 patients taking statins with no muscle symptoms.[29] Greater damage was seen in patients with muscle symptoms, whether or not the creatine kinase was elevated, and whether treatment was continuing or had been stopped for varying lengths of time. This suggests that the damage is not readily reversible.[30]

Clinical Implications

Statins work by inhibiting a critical enzymatic pathway and thus have many potential effects in addition to the reduction of serum cholesterol. **The full spectrum of statin related harms and their magnitude is still largely uncertain.** However, from this analysis it is clear that the magnitude of statin harms is greater with high doses than with low doses and that the added benefits of high doses is unlikely to exceed the magnitude of the harms in most if not all clinical settings.[14] Even for lower doses the magnitude of harms appears to be in the range of 1-2%, a range similar to the benefits of statins for primary prevention. Physicians must be vigilant in order to detect statin adverse effects as many of them are subtle. When statins interfere with exercise, the benefits of exercise are undermined.

Conclusions

- The action of statins to reduce many compounds in addition to cholesterol is problematic.
- Harms with statins are often subtle, usually dose related, sometimes serious and require vigilance to detect.
- The magnitude of most statins harms remains uncertain at this time.
- It is essential to weigh the potential benefits and the potential harms in all patients taking or being considered for statin therapy.

The draft of this Therapeutics Letter was submitted for review to 60 experts and primary care physicians in order to correct any inaccuracies and to ensure that the information is concise and relevant to clinicians.

The Therapeutics Letter presents critically appraised summary evidence primarily from controlled drug trials. Such evidence applies to patients similar to those involved in the trials, and may not be generalizable to every patient. We are committed to evaluate the effectiveness of our educational activities using the PharmaCare/PharmaNet databases without identifying individual physicians, pharmacies or patients. The Therapeutics Initiative is funded by the BC Ministry of Health through a grant to the University of BC. The Therapeutics Initiative provides evidence-based advice about drug therapy, and is not responsible for formulating or adjudicating provincial drug policies.

References

1. Golomb BA, Evans MA. Statin adverse effects: a review of the literature and evidence for a mitochondrial mechanism. Am J Cardiovasc Drugs 2008; 8:373-418.
2. Golomb BA, McGraw JJ, Evans MA, Dimsdale JE. Physician response to patient reports of adverse drug effects: implications for patient-targeted adverse effect surveillance. Drug Saf 2007; 30:669-675.
3. Mancini GBJ, Tashakkor AY, Baker S, et al. Diagnosis, prevention, and management of statin adverse effects and intolerance: Canadian Working Group Consensus update. Can J Cardiol 2013; 29:1553-1568.
4. Finegold JA, Manisty CH, Goldacre B, et al. What proportion of symptomatic side effects in patients taking statins are genuinely caused by the drug? Systematic review of randomized placebo-controlled trials to aid individual patient choice. Eur J Prevent Cardiolog 2014; 21:464-474.
5. Anonymous. MRC/BHF Heart Protection Study of cholesterol-lowering therapy and of antioxidant vitamin supplementation in a wide range of patients at increased risk of coronary heart disease death: early safety and efficacy experience. Eur Heart J 1999; 20:725–741.
6. Kurdyak PA, Juurlink DN, Mamdani MM. The effect of antidepressant warnings on prescribing trends in Ontario, Canada. Am J Public Health 2007; 97:750–754.
7. Canadian Adverse Reaction Newsletter Volume 12 · Number 1 · January 2002 http://www.hc-sc.gc.ca/dhp-mps/medeff/bulletin/carn-bcei_v12n1-eng.php#rhabdomyolysis_and_myopathy
8. Health Canada is advising Canadians about a possible association between Crestor® and rhabdomyolysis. http://www.healthycanadians.gc.ca/recall-alert-rappel-avis/hc-sc/2004/14251a-eng.php
9. Health Canada advises consumers about important safety information on statins http://www.healthycanadians.gc.ca/recall-alert-rappel-avis/hc-sc/2005/13698a-eng.php
10. Canadian Adverse Reaction Newsletter,Volume 15 • Issue 4 • October 2005 http://www.hc-sc.gc.ca/dhp-mps/medeff/bulletin/carn-bcei_v15n4-eng.php#a2
11. Canadian Adverse Reaction Newsletter, Volume 20 - Issue 4 - October 2010 http://www.hc-sc.gc.ca/dhp-mps/medeff/bulletin/carn-bcei_v20n4-eng.php#a1
12. FDA Drug Safety Communication: Important safety label changes to cholesterol-lowering statin drugs. February 28, 2012 found at: http://www.fda.gov/Drugs/DrugSafety/ucm293101.htm.
13. Silva M, Matthews ML, Jarvis C, et al. Meta-analysis of drug-induced adverse events associated with intensive-dose statin therapy. Clin Ther 2007; 29:253-260.
14. Preiss D, Seshasai SR, Welsh P, et al. Risk of incident diabetes with intensive-dose compared with moderate-dose statin therapy: a meta-analysis. *JAMA* 2011; 305:2556–2564.
15. Sattar N, Preiss D, Murray HM, et al. Statins and risk of incident diabetes: a collaborative meta-analysis of randomised statin trials. *Lancet* 2010; 375:735–742.
16. Golomb BA, Evans MA, Dimsdale JE, White HL. Effects of statin on energy and fatigue with exertion: Results from a randomized controlled trial. Arch Intern Med. 2012: doi:10.1001 /archinternmed.2012.2171
17. Mikus CR, Boyle LJ, Borengasser SJ, et al. Simvastatin impairs exercise training adaptations. J Am Coll Cardiol 2013; 62:709-714.
18. Amarenco P, Bogousslavsky J, Callahan A 3rd, et al. High-dose atorvastatin after stroke or transient ischemic attack. *N Engl J Med* 2006; 355:549–559.
19. Muldoon MF, Barger SD, Ryan CM, et al. Effects of lovastatin on cognitive function and psychological well-being. Am J Med 2000; 108:538-546.
20. Muldoon MF, Ryan CM, Sereika SM, et al. Randomized trial of the effects of simvastatin on cognitive functioning in hypercholesterolemic adults. Am J Med 2004; 117:823-829.
21. Golomb BA, Kwon EK, Criqui MH, Dimsdale JE. Simvastatin but not pravastatin affects sleep: findings from the UCSD Statin Study. Circulation 2007; 116:847.
22. Ridker PM, Danielson E, Fonseca FA, et al. Rosuvastatin to prevent vascular events in men and women with elevated C-reactive protein. *N Engl J Med* 2008; 359:2195–2207.
23. Dormuth CR, Hemmelgarn BR, Paterson JM et al. Use of high potency statins and rates of admission for acute kidney injury: multicenter, retrospective observational analysis of administrative databases. BMJ 2013; 346:f880.
24. Hippisley-Cox H, Coupland C. Unintended effects of statins in men and women in England and Wales: population based cohort study using the QResearch database. BMJ 2010; 340:c2197.
25. Mansi,I, Frei CR, Pugh MJ et al. Statins and Musculoskeletal Conditions, Arthropathies, and Injuries. JAMA Intern Med published online June 3, 2013. doi:10.1001/jamainternmed.2013.6184
26. Fernandez G, Spatz ES, Jablecki C, Phillips PS. Statin myopathy: a common dilemma not reflected in clinical trials. Cleve Clin J Med. 2011; 78:393-403.
27. Cohen JD, Brinton EA, Ito MK, Jacobson TA. Understanding Statin Use in America and Gaps in Patient Education (USAGE): an internet-based survey of 10,138 current and former statin users. J Clin Lipidol. 2012; 6:208-215.
28. Heran BS, Chen JMH, Ebrahim S, et al. Exercise-based cardiac rehabilitation for coronary heart disease. Cochrane Database of Systematic Reviews 2011, Issue 7. Art. No.: CD001800. DOI: 10.1002/14651858.CD001800.pub2.
29. Draeger, A, Monastyrskaya, K, Mohaupt, M, et al. Statin therapy induces ultrastructural damage in skeletal muscle in patients without myalgia. J Pathol. *2006;* 210:94–102.
30. Mohaupt MG, Karas RH, Babiychuk EB et al. Association between statin-associated myopathy and skeletal muscle damage. CMAJ 2009; 181:E11-E18.
