# Supplementary material for: Redesigning printed educational materials for primary care physicians: design improvements increase usability
Source: Implement Sci. 2015 Nov 4;10:156. doi: 10.1186/s13012-015-0339-5 (PMC4634785; doi:10.1186/s13012-015-0339-5)
Supplement: Additional file 2: — Redesigned Version of the Therapeutics Letter.ᅟ [file 13012_2015_339_MOESM2_ESM.pdf]

Statins: Proven and Associated Harms

THERAPEUTICS LETTER #89 | SPRING 2014

THERAPEUTICS INITIATIVE Evidence Based Drug Therapy

Key Messages

- Harms with statins are often subtle, usually dose-related, sometimes serious, and require vigilance to detect
- The magnitude of most statin harms remains uncertain at this time
- It is essential to weigh the potential benefits and the potential harms in all patients taking or being considered for statin therapy

This letter outlines proven and associated harms with statin use by examining evidence from systematic reviews and randomized controlled trials (proven harms) and observations studies, case series, and case reports (associated harms).

An Overview of the Evidence

Evidence from Systematic Reviews

Three systematic reviews have identified 4 adverse outcomes, outlined in Table 1. Silva and colleagues (2007) concluded that intensive dose therapy was associated with increased risk of statin-induced events.[13] Preiss and colleagues (2011) concluded that intensive-dose therapy was associated with increased risk of new-onset diabetes compared to low-dose.[14] Lastly, Sattar and colleagues (2010) found that statin therapy is associated with a slightly increased risk of development of diabetes.[15]

Evidence from Randomized Controlled Trials (RCTs)

Some statistically significant statin harms have been demonstrated in large RCTs, see Table 2. Others have been demonstrated in smaller RCTs designed to measure a specific effect. For example:

- The 2012 RCT showing that simvastatin and pravastatin significantly decrease energy and increase fatigue after exertion compared with placebo.[16]
- The 2013 RCT demonstrating that simvastatin significantly attenuates cardiorespiratory fitness as compared to placebo in overweight and obese patients.[17]

Evidence from Observational Studies

The magnitude of statin harms has also been estimated in large observational studies, as seen in Table 3.

Evidence from Case Series and Case Reports

A longer list of harms is documented in case series and case reports, as documented by Golomb and Evans[1], which include: peripheral neuropathy, sexual dysfunction, gynecomastia, irritability, aggression, behaviour change, memory loss, depression, psychosis, interstitial lung disease, heart failure, Parkinson syndrome, lupus-like syndrome, dermatomyositis, other auto-immune syndromes, pancreatitis, and others.

Table 1: Systematic Reviews

Outcomes	Dose	Effect Size: RR or OR (95% CI)	Number Needed to Harm
Withdrawal Due to Adverse Effects[13]	High vs Low	1.3 [1.2, 1.4]	47 (3.4 yr)
Muscle Damage (CK elevation > 10x normal)[13]	High vs Low	10.0 [1.3, 78]	1534 (3.4 yr)
Liver enzyme elevation[13]	High vs Low	4.8 [3.3, 6.2]	86 (3.4 yr)
Newly diagnosed diabetes[14]	High vs Low	1.2 [1.04, 1.22]	105 (4 yr)
Newly diagnosed diabetes[15]	All	1.09 [1.02, 1.17]	250 (4 yr)

High dose (80 mg simvastatin, 40-80 mg atorvastatin)
Low dose (20 mg simvastatin, 40 mg pravastatin, 10 mg atorvastatin)

Table 2: RCTs

Outcomes	Dose	Effect
Cognition[19, 20]	Low	Decrease
Energy[16]	Low	Decrease
Fatigue with exertion[16]	Low	Increase
Cardiorespiratory fitness[17]	Low	Decrease
Sleep quality[21]	Low	Decrease

Outcomes	Dose	Effect Size: RR or OR (95% CI)	Number Needed to Harm
Hemorrhagic stroke[18]	High [1]	1.7 [1.1, 2.6]	108 (5 yr)
Newly diagnosed diabetes[22]	High [2]	1.25 [1.05, 1.49]	167 (1.9 yr)

1 High dose (80 mg atorvastatin)
2 High dose (40 mg rosuvastatin)

Table 3: Large Observational Studies

Outcomes	Dose	Effect Size: RR or OR (95% CI)	Number Needed to Harm
Acute kidney injury[23]	High* vs Low	1.34 [1.25, 1.43]	1700 (0.25 yr)
Acute renal failure[24]	All doses vs no statin	1.6 [1.3, 1.9]	450 (5 yr)
Moderate or serious liver dysfunction[24]	All doses vs no statin	1.5 [1.4, 1.7]	150 (5 yr)
Moderate or serious myopathy[24]	All doses vs no statin	6.2 [5.2, 7.3]	100 (5 yr)
Musculoskeletal conditions[25]	All doses vs no statin	1.19 [1.08, 1.3]	48 (4.4 yr)
Cataracts[24]	All doses vs no statin	1.3 [1.26, 1.37]	50 (5 yr)

**High dose (≥10 mg rosuvastatin, ≥20 mg atorvastatin, ≥40 mg simvastatin)*

Muscle Symptoms:
The Most Common Statin Adverse Effect

- The incidence of muscle symptoms is low in RCTs[4] but higher in observational studies.[27]

- Muscle symptoms interfering with exercise and inhibition of cardiorespiratory fitness[17] are problematic because regular exercise is the best way for patients to prevent adverse cardiovascular events.[28]

- Minor muscle damage may be very prevalent as low level ultrastructural muscle damage was detectable in muscle biopsies from 10 of 14 patients taking statins with no muscle symptoms.[29]

- Damage may not be readily reversible.[30] Greater damage was seen in patients with muscle symptoms, whether or not the creatine kinase was elevated, and whether treatment was continuing or had been stopped for varying lengths of time.

- For an approach to dealing with patients with statin-related muscle symptoms, see Fernandez et al.[26]

Canadian and US Advisories on Statin-Related Harms

Since 2000, Health Canada has issued 5 Adverse Drug Reaction advisories related to statins:

1. Rhabdomyolysis and myopathy (January 2002)[7]
2. Crestor and rhabdomyolysis (November 2004)[8]
3. Existing medical conditions which may increase risk of statin-related muscle problems (July 2005)[9]
4. Statins and memory loss (October 2005)[10]
5. Statins and interstitial lung disease (October 2010)[11]

On February 28, 2012, the US FDA revised statin labels, warning of the potential for "generally non-serious and reversible cognitive side effects (memory loss, confusion, etc.) and reports of increased blood sugar and glycosylated hemoglobin (HbA1c) levels".[12]

Conclusions & Clinical Implications

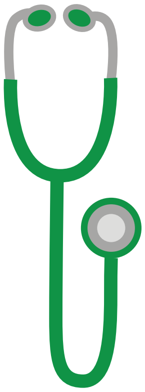

- The full spectrum of statin-related harms and their magnitude is still largely uncertain. However, from this analysis it is clear that the magnitude of harms from statins is greater with high doses than with low doses and that the added benefits of high doses is unlikely to exceed the magnitude of the harms in most if not all clinical settings.[14]

- Even for lower doses the magnitude of harms appears to be in the range of 1-2%, a range similar to the benefits of statins for primary prevention.

- When statins interfere with exercise, the benefits of exercise are undermined.

- Physicians must be vigilant in order to detect statin adverse effects as many of them are subtle.

- It is essential to weigh the potential benefits and the potential harms in all patients taking or being considered for statin therapy.

Why Aren't the Harms of Statins More Commonly Acknowledged?

Most of the literature on statins has focused on the benefits. As a result awareness of statin harms is low[2], and many specialists contend that statin harms are very unusual.[3] The reported incidence of common statin effects, such as muscle pain and weakening, is low in randomized trials but higher in studies of real world use.[1,4] To some extent this is explained by use of a 'run-in period' in some statin RCTs when all patients are exposed to the drug prior to randomization and only those tolerating the drug are randomized.[5] Warnings about statin-related harm issued by the US FDA or Health Canada are slow to be released, and as with past advisories have little impact.[6]

UBC

For the list of references, go to www.ti.ubc.ca/letter89

INFO@TI.UBC.CA

WWW.TI.UBC.CA
