# Supplementary material for: Redesigning printed educational materials for primary care physicians: design improvements increase usability
Source: Implement Sci. 2015 Nov 4;10:156. doi: 10.1186/s13012-015-0339-5 (PMC4634785; doi:10.1186/s13012-015-0339-5)
Supplement: Additional file 3: — Modified System Usability Scale. (DOCX 13.3 KB) [file 13012_2015_339_MOESM3_ESM.docx]

Additional file 3: Modified System Usability Scale

| **Therapeutics Letter Questions** |
| --- |
| I think that I would like to use this document frequently |
| I found this document unnecessarily complex |
| I thought this document was easy to use |
| I think that I would need the support of a technical person or content expert to be able to use this document |
| I found the various functions of this document (ex: the tables, boxes, graphics, etc.) were well integrated |
| I thought there was too much inconsistency in the format of this document |
| I would imagine that most people would learn to use this document very quickly |
| I found this document very cumbersome to use |
| I felt very confident using this document |
| I needed to learn a lot of things before I could get going with this document |
